# Diet and physical activity in people with intermediate cardiovascular risk and their relationship with the health-related quality of life: results from the MARK study

**DOI:** 10.1186/s12955-016-0572-x

**Published:** 2016-12-07

**Authors:** Natalia Sanchez-Aguadero, Rosario Alonso-Dominguez, Luis Garcia-Ortiz, Cristina Agudo-Conde, Carmela Rodriguez-Martin, Angela de Cabo-Laso, Benigna Sanchez-Salgado, Rafel Ramos, Jose A. Maderuelo-Fernandez, Manuel A. Gomez-Marcos, Jose I. Recio-Rodriguez, MARK Group, Rafel Ramos, Ruth Martí, Dídac Parramon, Anna Ponjoan, Miquel Quesada, Maria Garcia-Gil, Martina Sidera, Lourdes Camós, Fernando Montesinos, Ignacio Montoya, Carlos López, Anna Agell, Núria Pagès, Irina Gil, Anna Maria Castro, Fernando Rigo, Guillermo Frontera, Antònia Rotger, Natalia Feuerbach, Susana Pons, Natividad Garcia, John Guillaumet, Micaela Llull, Mercedes Gutierrez, Cristina Agudo-Conde, Leticia Gómez-Sanchez, Carmen Castaño-Sanchez, Carmela Rodriguez-Martín, Benigna Sanchez-Salgado, Angela de Cabo-Laso, Marta Gómez-Sánchez, Emiliano Rodriguez-Sanchez, Jose Angel Maderuelo-Fernandez, Emilio Ramos-Delgado, Carmen Patino-Alonso, Jose I. Recio-Rodriguez, Manuel A. Gomez-Marcos, Luis Garcia-Ortiz

**Affiliations:** 1 Primary Care Research Unit, The Alamedilla Health Center, Castilla and León Health Service (SACYL), Biomedical Research Institute of Salamanca (IBSAL), Spanish Network for Preventive Activities and Health Promotion (redIAPP), 37003 Salamanca, Spain; 2 Department of Biomedical and Diagnostic Sciences, University of Salamanca, Salamanca, Spain; 3 Research Unit Family Medicine, Girona. Jordi Gol Institute for Primary Care Research (IDIAP Jordi Gol), Translab Research Group. Medical Sciences Department, School of Medicine, University of Girona, Girona Biomedical Research Institute (IDIBGI), Dr. Trueta University Hospital, Catalonia, Spain; 4 Department of Medicine, University of Salamanca, Salamanca, Spain; 5 Department of Nursing and Physiotherapy, University of Salamanca, Salamanca, Spain

**Keywords:** Health-related quality of life, Life style, Food habits, Exercise

## Abstract

**Background:**

To analyze the interplay between diet, physical activity and health-related quality of life in a Spanish randomly selected sample of individuals attended in general practitioners offices with intermediate cardiovascular risk.

**Methods:**

This study analyzed 314 subjects, aged 35–74 years (50.6% women), from the MARK study, conducted in Spain. Health related quality of life was measured by the SF-12 questionnaire. The assessment of the lifestyles included the diet quality index, the adherence to the Mediterranean diet and the leisure time physical activity practice.

**Results:**

The highest values of health related quality of life were obtained in the area of vitality (51.05 ± 11.13), while the lowest were found in the general health (39.89 ± 8.85). In the multiple linear regression analysis, after adjustment for age, gender and other confounders, for each point of increase in the Mediterranean diet adherence score, there was an increase of 1.177 points in the mental component value (*p* < 0.01). Similarly, for each point of increase in the Diet Quality Index Score, there was an increase in the mental component of 0.553 (*p* < 0.05). Likewise, the physical activity was positively associated with the physical function and vitality (β = 0.090 and 0.087, (*p* < 0.01 and *p* < 0.05), respectively).

**Conclusions:**

In people with intermediate cardiovascular risk, better food habits and greater adherence to the Mediterranean diet are associated with higher scores on the mental component of quality of life. Likewise, increased physical activity is related with positive scores on the physical function.

**Electronic supplementary material:**

The online version of this article (doi:10.1186/s12955-016-0572-x) contains supplementary material, which is available to authorized users.

## Background

Self-reporting of health outcomes have increasing relevance to research, clinical practice and health planning [1, 2]. Analysis of quality of life provides complementary information to traditional health indicators based on morbidity and mortality [3] and it is a valid measurement for self-perceived mental and physical health status that is closely associated with cardiovascular disease and all-cause mortality [4, 5]. One of the most used instruments worldwide to measure the health related quality of life (HRQL) is the SF-36 questionnaire or its shorter version SF-12 that reduces the administration workload [3, 4].

Self-perceived health status has been previously associated with diet. Poor diet quality, characterized by low consumption of particular healthy foods or nutrients has been associated with low mental and physical health [5–9]. Furthermore, evidence from dietary intervention studies indicates an improvement of quality of life through healthy diets [10, 11]. It has been argued that the social and cultural aspects related to the Mediterranean diet may provide additional health benefits [12]. The PREDIMED study showed in a follow-up period of 4.8 years, that an energy-unrestricted Mediterranean diet which includes consumption of extra-virgin olive oil or nuts resulted in an absolute risk reduction of approximately 3 major cardiovascular events per 1000 person-year, for a relative risk reduction of approximately 30%. These results support the benefits of the Mediterranean diet for cardiovascular risk reduction [13].

Currently, physical inactivity is a major public health problem [14]. Exercise reduces mortality and lowers the risk of developing physical and mental chronic diseases [15]. Studies in the general population [16–19] have found a positive association between physical activity, vitality and mental health. This improves the overall quality of life [20, 21]. Similar results were found in special populations such as the elderly or the obese [22, 23].

The subgroup of patients with intermediate cardiovascular risk is the group in which the highest number of cardiovascular events occur [24], and it is known the association of these with a worse HRQL [25]. The European Guidelines on cardiovascular disease prevention in clinical practice highlights the importance in those subjects of promoting healthy lifestyle behaviour by tackling unhealthy lifestyles (e.g. poor-quality diet, physical inactivity, smoking) and by optimising risk factors [26, 27]. Some studies have analyzed the relationship between quality of life, exercise and Mediterranean diet in healthy subjects and individuals with various pathologies but few have focused their research on individuals with intermediate cardiovascular risk. Therefore, the purpose of this study is to analyze the interplay between diet, physical activity and health related quality of life in a Spanish randomly selected sample of individuals attended in general practitioners offices with intermediate cardiovascular risk.

## Methods

### Design

The MARK study [28] is a longitudinal study to evaluate if ankle-brachial index (ABI), measures of arterial stiffness (CAVI), postprandial glucose, glycosylated hemoglobin, self-measured blood pressure and the presence of comorbidities are independently associated with the incidence of vascular events and whether they can improve the predictive capacity of current risk equations in the intermediate risk population. The current study refers to the baseline visit. The second step will be 5- and 10-years follow up to evaluate cardiovascular morbidity and mortality.

### Study population

The study population comprised 500 subjects recruited in Salamanca, aged 35–74 years, with intermediate cardiovascular risk, defined as coronary risk 5–15% at 10 years according to the adaptation of the Framingham risk equation (REGICOR) [29], risk of cardiovascular mortality among 1–5% to 10 years according to the SCORE equation [30] or moderate risk according to the 2007 European Society of Hypertension guidelines for the management of arterial hypertension [31]. Terminal illness, institutionalization at the time of the appointment or personal history of atherosclerotic disease was excluded. Sample selection was performed by random sampling from the individuals attended for general practitioners at a health center that matched the inclusion criteria. The recruitment and data collection were carried out between July 2011 and June 2013. The current study analyzed 314 of these participants that were assessed for HRQL.

### Sample size calculation

The sample size was estimated to recognize as statistically significant a difference greater than or equal to 3.75 units on the SF-12 score between sedentary and active subjects (ratio 3:1). Accepting an alpha risk of 0.05 and a beta risk of 0.2 in a two-sided test, 78 subjects are necessary in first group and 234 in the second one. The common standard deviation is assumed to be 10. We anticipated a drop-out rate of 3.5%.

### Measurements

#### Assesment of the usual diet and the adherence to the Mediterranean diet

The usual diet was evaluated with the diet quality index (DQI) [32], a validated questionnaire about the frequency of consumption of 18 food groups divided into three categories. The first one included daily food consumption; less than, equal to or greater than 1 serving/day was scored with 1, 2 and 3 points, respectively; except for alcohol, in which case 3 points were assigned to the daily consumption of 1 alcoholic beverage and 1 point to an intake greater or lower than 1 per day. The second category, relating to weekly food consumption considered detrimental, was valuated with 2 points when the consumption was between 4 and 6 servings/week and 1 and 3 points when the intake was lower and upper, respectively. The third category included foods considered beneficial whose consumption between 2 and 3 times per week got 2 points, while the highest and lowest were scored with 3 and 1 point, respectively. Total score had a range from 18 to 54 points, with higher scores associated to a better diet quality. Adherence to the Mediterranean diet was collected with a short questionnaire previously validated, which provides 9 items relating to compliance with various aspects of the Mediterranean diet such as the consumption of olive oil, vegetables, fruit, nuts or white meat. Scores equal to or greater than 5 points are considered good compliance of the Mediterranean diet [33].

#### Assesment of the regular physical activity

Leisure time physical activity (LTPA) practice was collected with the short version of the Minnesota LTPA Questionnaire validated for Spanish men and women [34, 35]. The questionnaire was administered by trained interviewers colleting detailed information about physical activity (PA) during the preceding year, the number of times this activity was performed and the average duration of each activity on each occasion. Each PA has an intensity code based on the ratio between the metabolic rate during PA practice and the basal metabolic rate (MET). We assumed that 1 MET approximately corresponds to 1 kcal/min of energy expenditure. Therefore, we can calculate the total energy expenditure in leisure time of PA in kilocalories per week. Consumption of MET-min was estimated at week by multiplying the MET in physical activity for their duration (in minutes) and cumulative frequency in the month prior to the interview. We classified people according to energy expenditure in leisure time for 14 days in the following categories [36]: Very active: energy expenditure above 5000 METs-min/14 days; Active: energy expenditure between 3000 and 4999 METs-min/14 days; Moderately active: energy expenditure between 1250 and 2999 METs-min/14 days and Sedentary: less than 1250 METs-min/14 days energy expenditure.

#### Health related quality of life (HRQL)

HRQL was assessed with the Spanish version of the SF-12 v.2, which has been validated [3, 4]. The SF-12 is a shorter version of the SF-36 questionnaire [37], and includes 12 items, with 3 to 5 response categories on a Likert scale. The SF-12 questionnaire is self-administered and was developed to measure eight dimensions of HRQL: Physical Functioning, Role Physical, Body Pain, General Health, Vitality, Role Emotional, Social Functioning and Mental Health. These eight dimensions can be aggregated into two summary measures: a physical component summary (PCS-12) and a mental component summary (MCS-12). To estimate summary components of SF-12 (PCS-12 and MCS-12), we calculated the algebraic sum of the standardized scores of eight dimensions (z scores) weighted by weights. Physical and Mental Health Composite Scores (PCS-12 & MCS-12) are computed using the scores of the twelve questions and range from 0 to 100, where a zero score indicates the lowest level of health measured by the scales and 100 indicates the highest level of health [38]. The values are standardized to a US norm with a mean of 50 and a standard deviation of 10. Thus, the SF-12 summaries compare the scores for each study participant against the mean score in the population. A higher score in the PCS-12 or the MCS-12 corresponds to better health status. The two standardized summary scores provide a concise approximation of the physical and mental components of HRQL [38]. More details and a brief description of the components of the SF-12 questionnaire can be seen in Additional file 1: Table S1.

#### Definition of cardiovascular risk factors

Hypertension was defined as having one or more of the following conditions: physician-diagnosed hypertension, systolic blood pressure (BP) ≥ 140 mmHg, diastolic BP ≥ 90 mmHg and use of antihypertensive agents. Dyslipidemia was defined as having one or more of the following conditions: physician-diagnosed dyslipidemia, total cholesterol ≥ 250 mg/dl and use of lipid-lowering agents. Type 2 diabetes was defined as having one or more of the following conditions: physician-diagnosed type 2 diabetes, fasting plasma glucose (FPG) ≥ 126 mg/dl, HbA1c ≥ 6.5% and use of oral hypoglycemic agents. In relation to lifestyle habits, we classified the participants as non-smokers or current smokers and considered alcohol risk consumption when the intake was ≥ 290 g/week in males or ≥ 180 g/week in females.

#### Comorbidity study

The Charlson Comorbidity Index, which contains 19 comorbidity categories, was calculated. Each index category has an associated weight, taken from the original Charlson’s document [39], which is based on the adjusted mortality risk per year. The overall comorbidity score reflects the greater cumulative probability of mortality per year. The higher the score the more severe the comorbidity burden is.

#### Other variables

Clinical blood pressure (BP) determination involved three measurements of systolic blood pressure (SBP) and diastolic blood pressure (DBP), made with a validated sphygmomanometer (OMRON Model M10-IT) on the dominant arm of participants in the seated position after at least 5 min of rest with a cuff of appropriate size as determined by measurement of the upper arm circumference, using the average of the last two and following the recommendations of the European Society of Hypertension [40]. Body weight was determined on two occasions, with the subject wearing light clothing and no shoes, using a homologated electronic balance (Seca 770) properly calibrated (precision ±0.1 kg). Height was measured with subject standing barefoot, recording the average of two readings rounded to the nearest centimeter, using a portable system (Seca 222). Body mass index (BMI) was calculated as the weight (kg) divided by height squared (m^2^). Obesity was defined at values ≥30 kg/m^2^. Blood and urine tests were performed to evaluate lipids, glucose and kidney function to assess vascular risk. Other variables included sociodemographic variables and medical treatments.

### Statistical analysis

The results are expressed as mean ± standard deviation in quantitative variables or by the frequency distribution in the case of qualitative variables. Normality was assessed using the Kolmogorov-Smirnov test. The Chi-square test analyzed the association between independent qualitative variables. We used the Student’s *t* test for independent samples or Mann-Whitney test to compare the average of two groups. The relationship between quantitative variables were analyzed using the Pearson correlation coefficient. We performed three multiple linear regression analyses using the Multivariate General Linear Model (GLM), including as independent variables the mediterranean diet adherence, DQI and METS-min/week/100 (to facilitate the intrepretation). The eight SF-12 dimensions and standardized physical and mental component were the dependent variables. We performed a first model unadjusted and a second model adjusted for age, gender, hypertension, dyslipidemia and Charlson Comorbidity Index. For bilateral hypothesis contrasts, an alpha risk of 0.05 was set as the limit of statistical significance using SPSS v.21.0.

## Results

The study population consists of 314 participants with a mean age of 61.1 ± 8.4 years (50.6% women). The main risk factors included 86.6% with dyslipidemia, 75.8% with hypertension, 26.4% with obesity and 24.2% with Type 2 diabetes mellitus. With respect to lifestyle, 167 (53.2%) followed the Mediterranean diet, 78 (24.8%) were sedentary, 68 (21.7%) were current smokers at the time of interview, and 21 (6.7%) were at a high risk level of alcohol consumption. There were gender differences for physical activity and alcohol consumption, in favor of men. All characteristics of the study population are presented in Table 1.

**Table 1:** Clinical characteristics and lifestyles of the study population

	Global *n* = 314		Men *n* = 155 (49,4%)		Women *n* = 159 (50,6%)		
	Mean or number	SD or %	Mean or number	SD or %	Mean or number	SD or %	*p* value
Age (years)	61.1	8.4	60.0	8.9	62.2	7.9	0.019
Hypertension (n, %)	238	75.8	122	78.7	116	73	0.239
Type 2 Diabetes Mellitus (n, %)	76	24.2	44	28.4	32	20.1	0.113
Dyslipidemia (n, %)	272	86.6	132	85.2	140	88.1	0.509
Obesity (n, %)	83	26.4	39	25.2	44	27.7	0.701
Body mass index (Kg/m^2^)	28.1	4.3	28.2	3.6	28.0	4.9	0.721
Diet							
Adherence to the Mediterranean diet (n, %)	167	53.2	75	48.4	92	57.9	0.113
Mediterranean diet (total score)	5.5	1.5	5.4	1.5	5.6	1.5	0.243
Diet Quality Index (total score)	31.3	2.7	31.3	2.8	31.4	2.6	0.728
Physical activity							
METS-min/week	1773	1703	2211	2086	1346	1062	<0.001
Physical activity classification							
Sedentary (n, %)	78	24.8	31	20.0	47	29.6	<0.001
Moderately active (n, %)	90	28.7	41	26.5	49	30.8	
Active (n, %)	71	22.6	30	19.4	41	25.8	
Very active (n, %)	75	23.9	53	34.2	22	13.8	
Tobacco							
Smokers (n, %)	68	21.7	38	24.5	30	18.9	0.273
Alcohol							
Grames/week	73.1	109.9	116.4	134.9	30.9	50.7	<0.001
Risk consumption (n, %)	21	6.7	17	11.0	4	2.5	<0.001
Comorbidities							
Charlson Comorbidity Index	2.5	0.8	2.5	0.8	2.5	0.9	0.376

*MET* basal metabolic rate

Table 2 describes the mean scores in the quality of life questionnaire (SF-12). The highest values were obtained in the area of vitality (51.05 ± 11.13), while the lowest were found in the general health (39.89 ± 8.85). For all items, self-perception was greater in men (*p* < 0.05) except for the general health and vitality.

**Table 2:** Health-related quality of life (SF12)

	Global *n* = 314		Men *n* = 155 (49,4%)		Women *n* = 159 (50,6%)		
	Mean	SD	Mean	SD	Mean	SD	*p* value
Physical component	48.36	9.29	50.05	7.55	46.71	10.48	0.001
Mental component	49.47	10.48	51.36	9.74	47.64	10.88	0.002
Physical function	50.69	9.04	52.53	7.90	48.90	9.72	<0.001
Physical role	50.92	9.20	52.59	8.19	49.29	9.85	0.001
Bodily pain	49.84	11.65	53.23	9.27	46.54	12.77	<0.001
General health	39.89	8.85	40.63	8.51	39.16	9.13	0.142
Vitality	51.05	11.13	52.16	11.18	49.96	11.01	0.080
Social functioning	49.87	9.75	51.61	8.79	48.18	10.36	0.002
Emotional role	48.88	9.70	50.66	9.06	47.14	10.00	0.001
Mental health	49.68	10.45	52.46	10.03	46.98	10.18	<0.001

The METs-min/week were directly related to the physical and mental component (*r* = 0.141, *p* < 0.05) (*r* = 0.112, *p* < 0.05), respectively. This was more significant with physical function (*r* = 0.252, *p* < 0.01). It was also associated with bodily pain, vitality, as well as emotional and mental health (*p* < 0.05, all). The Mediterranean Diet (total score) was related to the mental component (*r* = 0.164, *p* < 0.01) as well as social functioning (*r* = 0.172, *p* < 0.01) and vitality (*r* = 0.122, *p* < 0.05). Moreover, the index of diet quality (DQI) was directly related to the mental component (*r* = 0.127, *p* < 0.05) and mental health (*r* = 0.121, *p* < 0.05) (Table 3).

**Table 3:** Bivariate correlations between health related quality of life and lifestyles

	Physical component	Mental component	Physical function	Physical role	Bodily pain	General health	Vitality	Social functioning	Emotional role	Mental health
Standardized METS^a^	0.141*	0.112*	0.252**	0.107	0.122*	0.104	0.117*	0.075	0.128*	0.141*
Mediterranean diet	−0.022	0.164**	−0.023	−0.083	−0.032	0.091	0.122*	0.172**	0.087	0.083
Diet quality index	−0.056	0.127*	0.004	−0.023	−0.047	0.051	0.014	0.078	0.073	0.121*

^a^ *MET* basal metabolic rate

*p < 0.05, **p < 0.01

In the multiple linear regression analysis (Table 4), after adjusting for age, gender, hypertension, dyslipidemia and Charlson Comorbidity Index, we found a 1.177 point increase in the mental component for each increase of 1 point in the Mediterranean diet adherence score (*p* < 0.01). Vitality and Social Functioning also kept these associations after the adjustment (β = 0.958 and 0.990, (*p* < 0.05 and *p* < 0.01), respectively). Furthermore, Mediterranean diet adherence score was inversely associated with Role Physical, regardless of the adjustment variables considered (β = −0.694, (*p* < 0.05)). Similarly, for each point of increase in the Diet Quality Index Score, there would be an increase in the mental health of 0.553 and in the mental component of 0.553 (*p* < 0.01 and *p* < 0.05, respectively). In contrast, after the adjustment, the physical activity was associated with the physical function and vitality (β = 0.090 and 0.087, (*p* < 0.01 and *p* < 0.05), respectively).

**Table 4:** Multiple regression analysis of lifestyles health-related quality of life

		Without adjustment				Adjusted for age, sex, hypertension, dyslipidemia and Charlson Comorbidity Index			
Independent variable	Dependent variable	β	95% CI^a^		*p* value	β	95% CI^a^		*p* value
Mediterranean diet	Physical component	−0.593	−1.285	0.099	0.093	−0,582	−1.273	0.109	0.098
	Mental component	1.209	0.437	1.982	**0.002**	1,177	0.415	1.939	**0.003**
	Physical function	−0.298	−0.974	0.378	0.386	−0.229	−0.900	0.442	0.502
	Role physical	−0.637	−1.322	0.048	0.068	−0.694	−1.372	−0.017	**0.045**
	Bodily pain	−0.508	−1.378	0.363	0.252	−0.499	−1.339	0.341	0.243
	General health	0.558	−0.102	1.217	0.097	0.500	−0.163	1.163	0.139
	Vitality	0.898	0.071	1.725	**0.033**	0.958	0.132	1.785	**0.023**
	Social functioning	1.037	0.316	1.758	**0.005**	0.990	0.278	1.702	**0.007**
	Role emotional	0.540	−0.183	1.263	0.143	0.527	−0.192	1.245	0.150
	Mental health	0.612	−0.168	1.391	0.124	0.572	−0.179	1.324	0.135
DQI^b^	Physical component	−0.175	−0.556	0.206	0.366	−0.223	−0.607	0.161	0.254
	Mental component	0.583	0.158	1.008	**0.007**	0.553	0.129	0.977	**0.011**
	Physical function	−0.017	−0.388	0.354	0.929	−0.042	−0.414	0.330	0.824
	Role physical	0.034	−0.343	0.412	0.858	−0.055	−0.433	0.323	0.774
	Bodily pain	−0.161	−0.638	0.317	0.508	−0.217	−0.683	0.249	0.360
	General health	0.275	−0.086	0.637	0.135	0.242	−0.126	0.609	0.197
	Vitality	0.159	−0.297	0.615	0.493	0.186	−0.275	0.648	0.428
	Social functioning	0.339	−0.059	0.737	0.095	0.281	−0.117	0.680	0.165
	Role emotional	0.342	−0.053	0.738	0.090	0.303	−0.095	0.702	0.135
	Mental health	0.606	0.183	1.030	**0.005**	0.553	0.139	0.966	**0.009**
METS^c^-min/week	Physical component	0.032	0.002	0.062	**0.038**	0.039	−0.025	0.103	0.233
	Mental component	0.029	−0.005	0.063	0.099	0.018	−0.053	0.089	0.623
	Physical function	0.054	0.025	0.083	**<0.001**	0.090	0.029	0.151	**0.004**
	Role physical	0.019	−0.011	0.049	0.212	0.005	−0.068	0.058	0.870
	Bodily pain	0.030	−0.008	0.068	0.122	−0.003	−0.080	0.075	0.949
	General health	0.024	−0.005	0.053	0.106	0.039	−0.022	0.100	0.212
	Vitality	0.047	0.011	0.083	**0.011**	0.087	0.010	0.163	**0.026**
	Social functioning	0.015	−0.017	0.047	0.354	−0.009	−0.075	0.058	0.793
	Role emotional	0.030	−0.002	0.062	0.063	0.028	−0.038	0.094	0.408
	Mental health	0.038	0.004	0.072	**0.027**	0.017	−0.053	0.086	0.636

^a^ *CI* confidence interval, ^b^ *DQI* diet quality index, ^c^ *MET* basal metabolic rate

Bold data reflect *p* values <0.05

## Discussion

Food habits are related to the mental component of quality of life while physical activity is related to the physical function in a large sample of subjects with intermediate cardiovascular risk. Better food habits—characterized by greater adherence to the Mediterranean diet and a higher score on the diet quality index (DQI)—had the best scores on the mental component, vitality and social function. Likewise, increased physical activity as evaluated with the short Minnesota questionnaire is related to better physical function. To our knowledge, these are the first results that the quality of life is associated with lifestyles related to diet and physical exercise in a sample of adults with intermediate cardiovascular risk. This population has a privileged position to be the target in changing lifestyles that aim at preventing cardiovascular disease and improving quality of life. This relationship (between lifestyles and HRQL) has been shown to exist in other populations but not in subjects with an intermediate CVR [41–43].

Our data indicate that the perceived quality of life could be influenced by physical activity. We found a direct association that occurs for physical and mental components and for the majority of dimensions. However, after adjustment for confounders, only the physical function and vitality maintained statistical significance. These findings are consistent with those published by Kovacs et al. [17] in elderly women. After participating in an adapted physical activity program, there was a significant increase in the dimensions of physical function and vitality. Similarly, the comprehensive review of Vagetti et al. [44] indicated a positive association between physical activity and quality of life in the elderly that was consistently associated with functional ability and vitality, among other domains. A cross-sectional study of adults with obesity conducted by Jepsen et al. [23] prior to a lifestyle intervention showed a significant association with general health. In contrast, a randomized controlled clinical trial of inactive persons [16] with a program to promote physical activity as an intervention, found no statistically significant changes between the experimental and control group in regard to the quality of life related to health. Other authors have evaluated the quality of life with specific tools for their study population (elderly, obese and patients with cystic fibrosis) [22, 45, 46]. Similar to our work, they found that physical activity is directly associated with quality of life. Our results would support the idea that physical activity has implications on quality of life related to health to the extent that it might promote individual autonomy through positive changes in dimensions such as the physical functioning.

Our results support the findings of other studies [42] suggesting a beneficial association of DM with quality of life. However, we include a novelty little studied, the relationship between the quality of food, as measured by the DQI, with the quality of life. The DQI was created to rapidly capture quality of diet based on food intake. Diet quality indexes address the diet’s complexity and are calculated by a combination of foods and/or nutrients with a total score depicting overall diet quality [47]. In our study population, greater adherence to the Mediterranean diet was associated with higher scores on the SF-12 mental component, social functioning and vitality. Similarly, the diet quality index (DQI) showed an association with the mental component. Adopting a MD in adulthood reduces chronic disease burden and all-cause mortality [48]. Recent studies show that better adherence to the Mediterranean diet is associated with lower cardiovascular disease [13], lower risk of metabolic syndrome [49], better cognitive performance [50] and has a favourable role on the prevention of colorectal cancer [51]. These data are consistent with those published by Henriquez Sánchez et al., [52]. In a cohort study of Spanish university graduates, a positive association was observed between adherence to the Mediterranean diet and vitality (β = 3.38; 95% CI = 1.68–5.07), physical function (β = 2.13; 95% CI = 1.15–3.11) and general health (β = 2.84; 95% CI = 1.16–4.51). Likewise, Bonaccio et al. [53] showed that adherence to the Mediterranean diet was positively associated with dimensions of mental health (vitality, social function) and physical health (physical functioning, role limitations due to physical and general health), but not pain. Furthermore, Muñoz [54] included multiple confounders, but still found the same result—adherence to the Mediterranean diet was associated with higher levels of perceived health, both physically and mentally—.

The study by Ruano et al. [55] compared the quality of life in the Western diet (high consumption of fast food, red meat, and industrial pastry) with the Mediterranean Diet. Values for vitality, mental health, physical functioning, bodily pain and general health were significantly better in the population with greater adherence to the Mediterranean Diet. Moreover, another study published by Ruano et al. [56], linked the consumption of fat to quality of life and showed a significant inverse association between consumption of saturated fatty acids and the mental dimensions (vitality, social functioning and emotional role) and physical function (physical role and general health). Therefore, our results are in line with previous studies—a greater adherence to the Mediterranean diet is associated with higher scores on the mental dimension in the quality of life questionnaire (SF-12)—.

The main limitation of this study is its cross-sectional design that prevents any causal relationship between quality of life and lifestyles to be analyzed. The design (cross-sectional study) and the questionnaire used to evaluate the adherence to the MD do not allow to assess retrospectively adherence to this dietary pattern nor its temporal association with HRQL. Another limitation is that the quality of life questionnaire used was self-reported. However, this questionnaire has been previously used in studies of similar characteristics.

## Conclusion

In people with intermediate cardiovascular risk, better food habits and greater adherence to the Mediterranean diet are associated with higher scores on the mental component of quality of life. Likewise, increased physical activity is related with positive scores on the physical function.

## Additional file

Additional file 1: Table S1. Brief description of the components of the SF-12 questionnaire. (DOCX 12 kb)
