# Supplementary material for: Diet and physical activity in people with intermediate cardiovascular risk and their relationship with the health-related quality of life: results from the MARK study
Source: Health Qual Life Outcomes. 2016 Dec 7;14:169. doi: 10.1186/s12955-016-0572-x (PMC5142320; doi:10.1186/s12955-016-0572-x)
Supplement: Additional file 1: Table S1. — Brief description of the components of the SF-12 questionnaire. (DOCX 12 kb) [file 12955_2016_572_MOESM1_ESM.docx]

| **Table S1. Brief description of the components of the SF-12 questionnaire.** | |
| --- | --- |
| **Physical function** | Extent to which poor health limits physical activities of daily living: personal care, walking, climbing stairs, picking up or carrying loads, and make moderate to intense efforts. |
| **Physical role** | Extent to which poor health interferes with work and other daily activities, resulting in a lower yield of the desired, or limiting the types of activities that can be performed or the difficulty of them. |
| **Bodily pain** | Measurement of the intensity of pain experienced and its effect on the normal work and home activities. |
| **General health** | Health status, including the current situation and future perspectives and the resistance to illness. |
| **Vitality** | Feeling of energy and vitality, against fatigue and discouragement. |
| **Social functioning** | Extent to which physical or emotional problems derived from the lack of health interferes with the normal social life. |
| **Emotional role** | Extent to which emotional problems affect work and other daily activities, considering reducing the time spent, decreased working efficiency and dedication at work. |
| **Mental health** | Assessment of general mental health, considering depression, anxiety, self-control, and general well-being. |
| Gac Sanit 2005, 19(2):135-150. | |
